# Transcatheter Mitral Edge-to-Edge Repair: A Selection of the Mitral Regurgitation Subtype and Definition of the Optimal Time for Intervention

**DOI:** 10.31083/RCM44073

**Published:** 2025-12-04

**Authors:** Sara Amicone, Jessica Zannoni, Marta Barletta, Chiara Mainardi, Elena Cozza, Arianna Grelli, Alessandro Vella, Giorgia Marsili, Maurizio Tusa

**Affiliations:** ^1^Department of Medical and Surgical Sciences (DIMEC), Alma Mater Studiorum, University of Bologna, 40126 Bologna, Italy; ^2^Cardiovascular Division, Morgagni-Pierantoni University Hospital, 47121 Forlì, Italy; ^3^Clinical and Interventional Cardiology Department, IRCCS Policlinico San Donato, 20097 San Donato Milanese, Italy; ^4^Department of Cardiology, Tor Vergata Hospital of Rome, University of Rome “Tor Vergata”, 00133 Rome, Italy

**Keywords:** mitral regurgitation, primary mitral regurgitation, secondary mitral regurgitation, degenerative mitral regurgitation, functional mitral regurgitation, transcatheter edge-to-edge repair

## Abstract

In the era of mitral transcatheter edge-to-edge repair (M-TEER), growing evidence continues to support a shift from a binary classification of mitral regurgitation (MR) into primary and secondary forms toward a more refined, subtype-based approach. Additionally, anatomical and pathophysiological heterogeneity significantly influences procedural complexity, durability of repair, and clinical outcomes within both primary and secondary MR. Furthermore, recent trials suggest that the timing of the intervention is as critical as patient anatomy; delaying treatment until advanced ventricular remodeling has occurred may limit the benefits of MR reduction. Moreover, long-term data on durability and device-failure management remain limited, particularly in secondary MR, where the progression of the underlying cardiomyopathy largely determines the outcomes. Thus, this review underscores how integrating MR subtyping with intervention strategies may influence patient selection and highlights the need for future research to adopt a more individualized, mechanism-driven approach.

## 1. Introduction

Mitral regurgitation (MR) is one of the most common valvular heart diseases worldwide, with an estimated prevalence of 2–3% in the general population. This figure increases significantly with age, reaching over 13% in individuals older than 75 years [1].

In industrialized countries, the most frequent aetiologies include degenerative causes such as mitral valve prolapse and ischemic MR secondary to coronary artery disease. In contrast, rheumatic heart disease remains a leading cause in developing regions [2].

MR is generally classified as primary (organic), resulting from intrinsic abnormalities of the mitral valve apparatus, or secondary (functional), typically due to left ventricular dilation or dysfunction, or to atrial dilatation leading to annular dilation and leaflet malposition. Irrespective of its aetiologies, severe MR carries a poor prognosis when left untreated. Data from the Euro Heart Survey revealed that approximately 50% of patients with severe MR were not referred for surgical treatment, often due to age, comorbidities, or ventricular dysfunction, and these patients faced a one-year mortality risk nearly 60% higher than those who underwent intervention [3].

In order to address this substantial treatment gap, especially in high-risk or inoperable patients, the mitral transcatheter edge-to-edge repair (M-TEER) technique has emerged as a less invasive alternative to surgery.

Conceptually inspired by the surgical Alfieri stitch, which approximates the anterior and posterior mitral leaflets at the site of regurgitation to create a double-orifice valve and reduce MR, this approach has, in recent years, become a valid substitute for surgery in high-risk patients. Consequently, more than 200,000 patients have now been treated with this technique worldwide.

The objective of this review is to provide a comprehensive and up-to-date overview of the clinical evidence supporting M-TEER as a treatment for MR. The text places emphasis on the fundamental differences between various subtypes of primary and secondary MR. The distinguishing characteristics of these subtypes are defined by their distinct pathophysiological mechanisms and therapeutic responses. These differences significantly influence patient selection, procedural strategy, and clinical outcomes. Furthermore, the review suggests that the temporal aspect of intervention has emerged as a pivotal factor in optimising treatment outcomes, highlighting the ongoing challenge of determining the appropriate timing for surgical or transcatheter intervention.

## 2. M-TEER: From First-in-Human Use to Current Guidelines

**Fig. 1.**
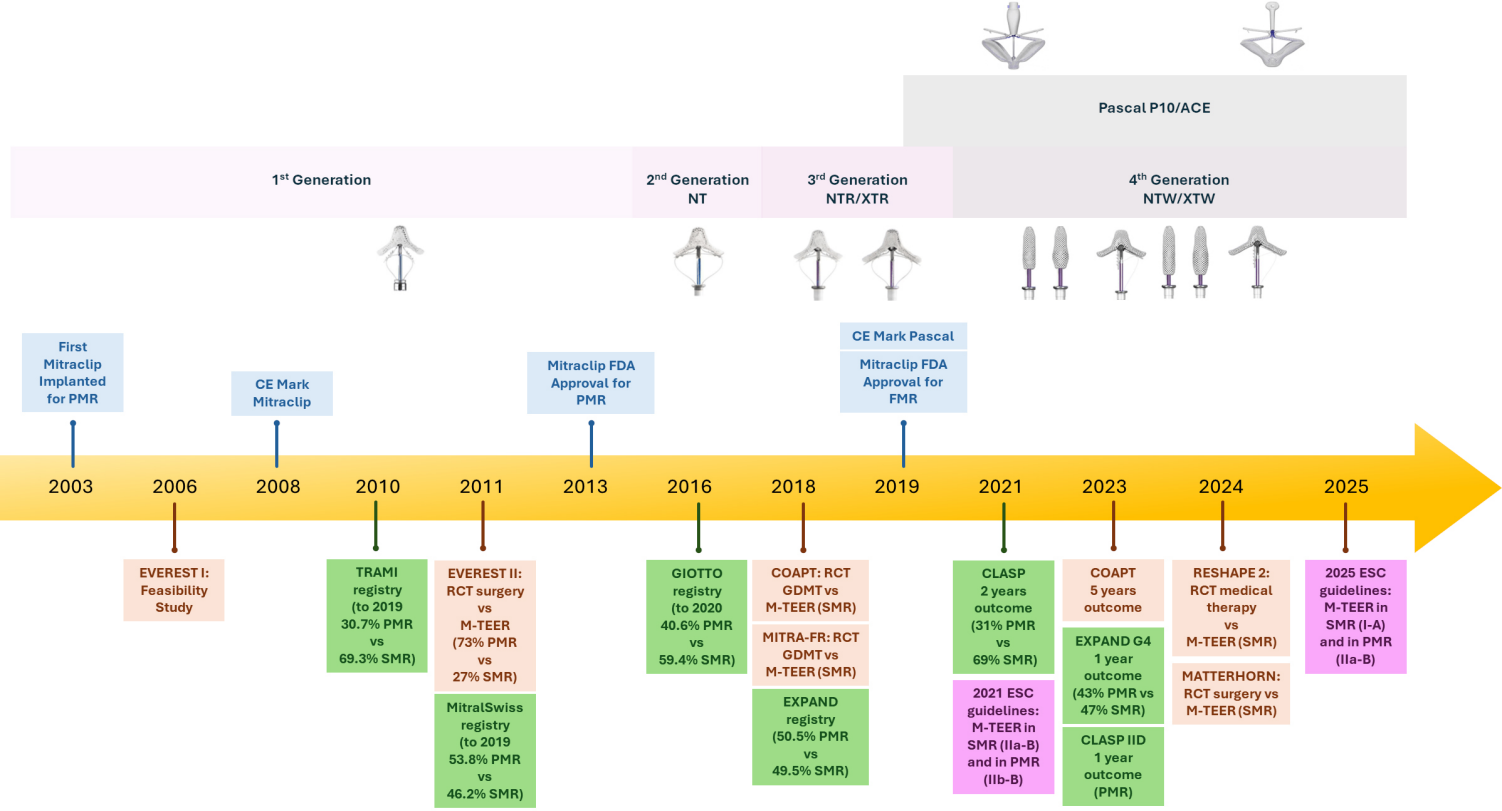
**Timeline of mitral transcatheter edge-to-edge repair (M-TEER) device development and major clinical evidence**. The upper section shows the generational evolution of the MitraClip (1st to 4th generation) and PASCAL systems. The lower section highlights key randomized controlled trials (RCTs) and real-world registries assessing M-TEER both in primary (PMR) and secondary (SMR) mitral regurgitation. Regulatory milestones (CE mark and FDA approvals) are also indicated. *Abbreviations*: PMR, primary mitral regurgitation; SMR, secondary mitral regurgitation; GDMT, guideline-directed medical therapy; ESC, European Society of Cardiology; FDA, Food and Drug Administration; CE, Conformité Européenne; EVEREST, Endovascular Valve Edge-to-Edge Repair Study; TRAMI, Transcatheter Mitral Valve Interventions; GIOTTO, Italian Society of Interventional Cardiology (GIse) registry Of Transcatheter treatment of mitral valve regurgitaTiOn; COAPT, Cardiovascular Outcomes Assessment of the MitraClip Percutaneous Therapy for Heart Failure Patients with Functional Mitral Regurgitation; MITRA-FR, Percutaneous Repair With the MitraClip Device for Severe Functional/Secondary Mitral Regurgitation; CLASP, Edwards PASCAL TrAnScatheter Mitral Valve RePair System.

The evolution of M-TEER into a guideline-endorsed therapy began with a series of pivotal milestones, illustrated in Fig. 1. The first-in-human experience of M-TEER took place in 2003. Currently, two systems are approved in Europe and America for M-TEER: the MitraClip system and the PASCAL system. The PASCAL system (Edwards Lifesciences, Irvine, California) received its Conformité Européenne (CE) Mark approval in February 2019. The CLASP IID (Edwards PASCAL TrAnScatheter Valve RePair System Pivotal Clinical) trial confirmed the safety and efficacy of the PASCAL system, achieving non-inferiority endpoints compared with the MitraClip system and, therefore, broadening transcatheter treatment options for patients with significant symptomatic primary mitral regurgitation (PMR) who are at prohibitive surgical risk [4].

The MitraClip system (Abbott Vascular, Santa Clara, CA, USA) was approved by the CE in 2008 and by the Food and Drug Administration (FDA) in 2013 for PMR and in 2019 for secondary mitral regurgitation (SMR), primarily based on findings from the EVEREST II (Endovascular Valve Edge-to-Edge Repair Study) trial [5]. Approximately 73% of patients in the EVEREST II had PMR. The study demonstrated that surgical intervention exhibited superiority in terms of a composite efficacy endpoint (freedom from death, surgery for mitral-valve dysfunction, and grade 3+ or 4+ mitral regurgitation) achieved in 73% of surgical patients, as opposed to 55% of patients treated with MitraClip at 12 months (*p* = 0.007). This result was confirmed at the 5-year follow-up [6]. This difference was largely driven by a higher rate of residual MR and reintervention in the MitraClip group within the first six months. Notably, M-TEER was associated with significantly fewer bleeding events and peri-procedural complications, suggesting its clinical value in high-risk patients.

These findings laid the foundation for the first appearance of M-TEER in the 2012 European Society of Cardiology (ESC) guidelines, where the technique was recognized as an alternative for symptomatic patients with severe primary or secondary MR who were anatomically suitable and deemed inoperable or at excessive surgical risk by the Heart Team (class of recommendation IIb, level of evidence C) [7].

The subgroup analyses from EVEREST II provided important insights that would guide subsequent studies and guideline updates. While surgery remained clearly superior in PMR, the SMR subgroup appeared to derive comparable benefits from the percutaneous approach, though with a wide confidence interval, with a statistically significant interaction suggesting a potentially more favourable effect of M-TEER in the SMR cohort, supporting future randomized trials (RCTs) specifically focused on this population.

Consequently, in 2018, the role of M-TEER in patients with SMR was studied for the first time in two pivotal RCTs: MITRA-FR (Percutaneous Repair with the MitraClip Device for Severe Functional/Secondary Mitral Regurgitation) and COAPT (Cardiovascular Outcomes Assessment of the MitraClip Percutaneous Therapy for Heart Failure Patients with Functional Mitral Regurgitation) [8, 9]. Both studies evaluated the addition of M-TEER to optimal guideline-directed medical therapy (GDMT) in patients with heart failure and SMR. Despite similar procedural approaches, the trials generated markedly divergent results, sparking extensive discussion in the field. In MITRA-FR, M-TEER failed to show a significant clinical benefit over GDMT alone in terms of mortality or heart failure hospitalizations [8]. In contrast, COAPT demonstrated a clear and sustained advantage of M-TEER: patients undergoing the procedure experienced a reduction in heart failure hospitalizations and in all-cause mortality at two years [9]. Several hypotheses have been proposed to explain this discrepancy. Key differences include stricter patient selection in COAPT, a greater severity of MR relative to LV dilation, and lower procedural complication rates.

However, in recognition of the COAPT findings, the CE mark and U.S. FDA approval for the treatment of SMR were granted in 2019, further solidifying the clinical utility of M-TEER in this patient population. Subsequently, the 2021 ESC guidelines upgraded the recommendation for M-TEER in selected patients with SMR who meet COAPT-like criteria to class of recommendation IIa, level of evidence B [10].

In 2024, the RESHAPE-HF2 trial (A Randomized Study of the MitraClip Device in Heart Failure Patients with Clinically Significant Functional Mitral Regurgitation) aimed to definitively address the discrepancies between MITRA-FR and COAPT. Indeed, the RESHAPE-HF2 trial enrolled 505 patients with heart failure (left ventricular ejection fraction (LVEF) 20–50%) and moderate-to-severe or severe SMR (Effective Regurgitant Orifice Area >0.2 cm^2^), randomized to GDMT alone or combined with M-TEER [11]. At 2 years, M-TEER significantly reduced the composite of cardiovascular death and heart failure (HF) hospitalizations, recurrent HF admissions, and improved quality of life, although no reduction in all-cause mortality was observed [11]. Compared with MITRA-FR, but in line with COAPT, RESHAPE-HF2 excluded patients with severe right ventricular dysfunction, tricuspid regurgitation, or hemodynamic instability. Moreover, enrolled patients had less advanced disease (lower N-terminal pro-B-type natriuretic peptide(NT-pro-BNP), better renal function, less severe MR, and higher rates of GDMT). A prespecified subanalysis showed that those with a recent HF hospitalization derived the greatest prognostic benefit. Overall, RESHAPE-HF2 demonstrates that in clinically stable, symptomatic patients without right heart involvement, M-TEER provides significant prognostic benefit even in non-severe MR, supporting the value of an earlier intervention strategy [11].

Moreover, in the simultaneously published study-level meta-analysis pooling RESHAPE-HF2, COAPT, and MITRA-FR, M-TEER was associated with a clear reduction in HF hospitalizations at 2 years after randomization, along with a trend toward improved survival, although the mortality benefit did not reach statistical significance [12].

Ultimately, MATTERHORN was the first trial to randomize symptomatic patients with SMR, already receiving maximally tolerated GDMT, to either M-TEER or MV surgery in a noninferiority design. M-TEER proved noninferior to surgery with respect to the composite endpoint of death, heart failure rehospitalization, stroke, reintervention, or left ventricular assist device implantation at one year [13]. However, the non-inferiority design, relatively short 12-month follow-up, broad non-inferiority margin, and variability in the surgical control arm may influence the interpretation of the MATTERHORN results. First of all, the short-term nature of the primary endpoint may not fully capture the long-term benefits or risks of MV surgery, which is known to have an initial hazard phase but potential late advantages.

In light of the aforementioned RCTs, according to the 2025 European guideline, M-TEER currently carries a class I recommendation (level of evidence A) for patients with ventricular SMR who remain symptomatic despite GDMT and who meet technical and clinical feasibility criteria. It is further assigned a class IIb recommendation (level of evidence B) in patients with atrial SMR at high surgical risk, and a class IIa recommendation (level of evidence B) in symptomatic patients with PMR at high surgical risk and with favourable anatomy for M-TEER [14].

In contrast, the 2020 American College of Cardiology (ACC) and the American Heart Association (AHA) guidelines suggest a Class IIa recommendation (level of evidence B) for M-TEER in symptomatic patients diagnosed with severe SMR despite GDMT, provided that favourable anatomy is present and the patient is categorised as high or prohibitive surgical risk. For PMR, surgery remains the treatment of choice (class I, level of evidence B), while M-TEER holds a class IIa recommendation (level of evidence B) for high-risk symptomatic patients with suitable valve anatomy [15]. However, it should be noted that American guidelines were published before the publication of the RESHAPE-HF2 results and before the 5-year COAPT follow-up.

**Key message**: In recent years, M-TEER has evolved into a valuable therapy for both primary and secondary MR. Recent data confirm the safety and efficacy of the procedure, especially in the context of SMR. The expanding role of M-TEER is now reflected in the latest European guidelines, which carry out, for selected patients, a class I recommendation in SMR and a class IIa recommendation in PMR.

## 3 Primary Mitral Regurgitation

### 3.1 WHAT: What Primary MR are We Treating?

**Fig. 2.**
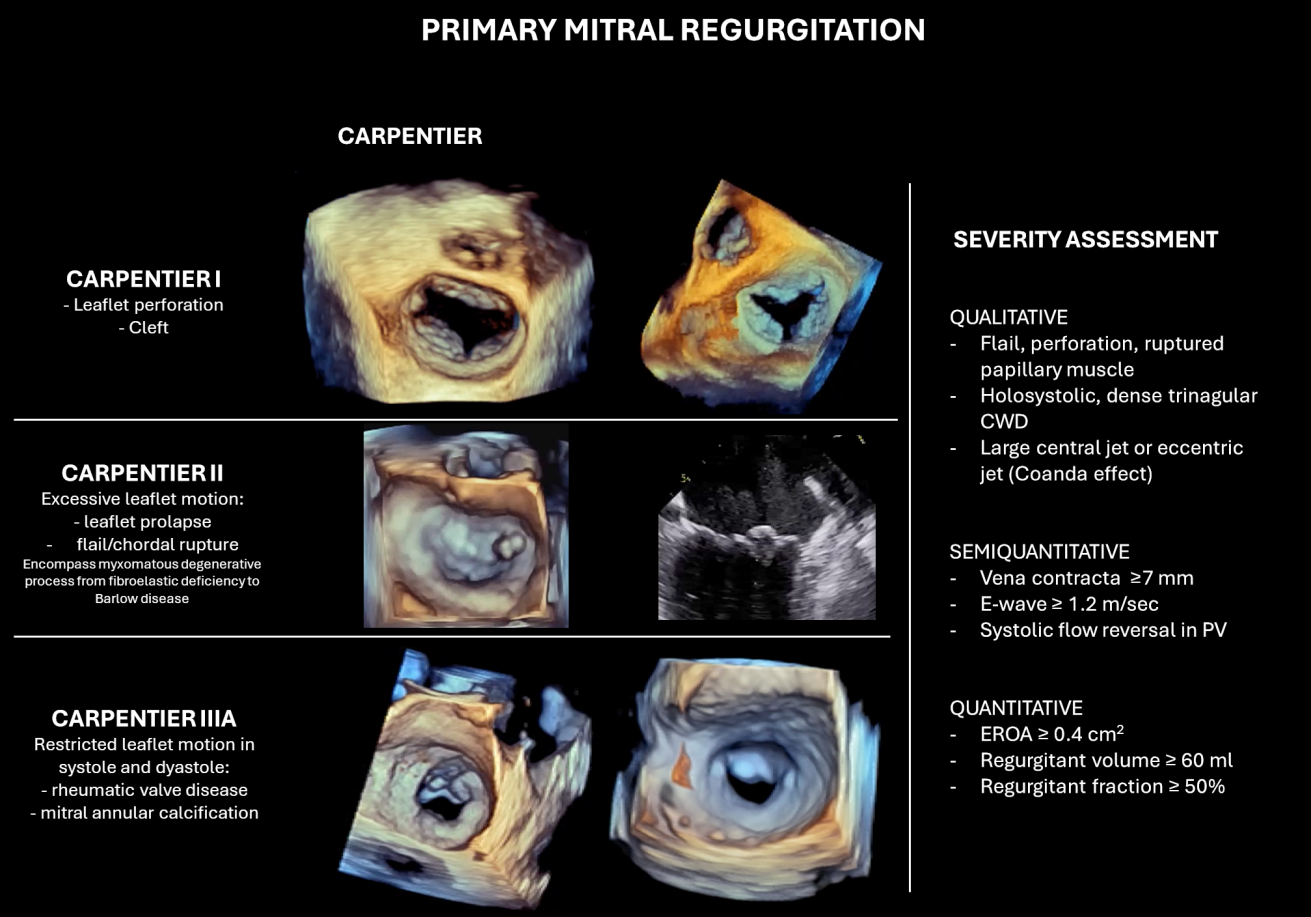
**Classification and echocardiographic severity assessment of primary mitral regurgitation (PMR)**. The left panel illustrates Carpentier’s classification in PMR. The right panel summarizes qualitative, semi-quantitative, and quantitative parameters used for MR grading. Abbreviations: CWD, continuous wave Doppler; PV, pulmonary vein; EROA, effective regurgitant orifice area.

PMR is associated with structural abnormalities of the mitral valve apparatus, particularly involving the leaflets or chordae tendineae. According to the Carpentier classification, type I PMR is characterised by normal leaflet size and motion, with the MR due to leaflet perforation or clefts. Type II is defined by excessive leaflet motion, typically resulting from leaflet prolapse or chordal rupture. Carpentier type IIIa MR describes leaflet restriction both in diastole and systole, most commonly seen in rheumatic disease (Fig. 2). However, the predominant aetiology of PMR, in developed countries, is myxomatous degeneration of the MV, which encompasses mitral valve prolapse and, in such cases, flail leaflet segments.

Degenerative MV disease presents in two main phenotypes that represent opposite ends of a pathological spectrum: fibroelastic deficiency and Barlow’s disease (BD).

- Fibroelastic deficiency is more frequently observed in patients over 60 years old. The condition is characterised by a deficiency in connective tissue, which results in thinning of the leaflet tissue. Rupture of thin, deficient chords is the usual mechanism of regurgitation in these patients.
- In contrast, BD tends to affect younger individuals between 40 and 60 years of age. This form is distinguished by diffuse myxomatous degeneration, with redundant, thickened, and elongated mitral leaflets and chordae. Multiple scallops of both anterior and posterior leaflets may prolapse or flail into the left atrium during systole, leading to severe and complex regurgitation.

It is important to note that most patients with degenerative MV disease exhibit features that lie somewhere between these two archetypes, rather than fitting neatly into either category.

Other PMR aetiologies include leaflet perforation, cleft, rheumatic disease, radiation, connective tissue disease, and mitral annulus calcification.

Correct identification of the underlying mechanisms of MR is imperative for selecting appropriate candidates for M-TEER. It is known that specific anatomical characteristics, including leaflet clefts, extensive leaflet calcification, or restricted leaflet motion resulting from rheumatic disease, are generally considered contraindications or high-risk scenarios for M-TEER. Among degenerative forms, BD with redundant, thickened, and elongated leaflets can complicate both grasping and correct positioning of the device. Furthermore, the excessive motion of the leaflets and annular dilation in BD increase the risk of higher residual MR. Conversely, patients demonstrating fibroelastic deficiency with an isolated flail segment, particularly when located on the middle scallop of the posterior leaflet (P2), exhibit favourable anatomical characteristics for M-TEER.

**Key message**: PMR encompasses a spectrum of degenerative valve diseases, primarily fibroelastic deficiency and BD, each with distinct anatomical and clinical features. Accurate mechanism-based diagnosis is imperative, given the significant influence of leaflet morphology on M-TEER outcomes. Candidates deemed suitable for the procedure typically exhibit isolated flail segments, while BD with multiscallop prolapse presents a greater procedural challenge.

### 3.2 WHO: Who Should We Treat?

In PMR, severity is defined by a combination of echocardiographic measurements, with particular attention to integrative findings that reflect the volume and impact of regurgitation.

The diagnosis of severe MR is made through qualitative and quantitative assessment, with the quantitative criteria set as follows:

- Effective regurgitant orifice area (EROA) ≥0.40 cm^2^.
- Regurgitant volume (RVol) ≥60 mL/beat.
- Regurgitant fraction ≥50%.

Additional qualitative and semi-qualitative supporting findings include flail and corde rupture, leaflet perforation, cleft, vena contracta ≥0.7 cm (≥0.8 cm for biplane), large central or eccentric jet occupying more than 50% of the left atrium, systolic flow reversal in the pulmonary veins, and signs of left atrial and ventricular enlargement, which suggest chronic volume overload (Fig. 2).

An accurate classification of MR severity is essential not only for decision-making regarding surgery or M-TEER but also for identifying the optimal timing of intervention before the onset of irreversible ventricular dysfunction or adverse clinical outcomes.

In accordance with the recently published ESC guidelines, surgical MV repair remains the first-line treatment for patients with symptomatic severe PMR who are not at high operative risk (class of recommendation I, level of evidence B). Surgery should also be strongly considered in asymptomatic patients who show early signs of left ventricular dysfunction, such as a LVEF ≤60% or a left ventricular end-systolic diameter (LVESD) ≥40 mm (class of recommendation I, level of evidence B) [14].

In case of severe MR, the total stroke volume incorporates both the volume ejected into the aorta and the volume that regurgitates back into the left atrium. As LVEF is reflective of total output, it may appear preserved despite underlying LV dysfunction, thereby underestimating the true severity of systolic impairment. Moreover, the elimination of volume overload post-surgery frequently results in a postoperative decline in LVEF, which may reveal undetected preexisting dysfunction. This is supported by evidence indicating that delaying intervention until LVEF falls below 60% is associated with increased long-term mortality [16]. Furthermore, LVESD is contingent upon contractility and afterload, yet remains independent of preload. Consequently, it is a valuable metric for assessing function in MR and has been associated with worse outcomes [17].

Historically, asymptomatic patients with preserved LV function (LVEF >60%, LVESD <40 mm) were monitored conservatively. However, the presence of pulmonary hypertension (pulmonary artery systolic pressure (PASP) at rest >50 mmHg), atrial fibrillation secondary to MR, and significant atrial dilatation (LAVI ≥60 mL/m^2^) serve as predictors of adverse long-term outcomes [18]. Consequently, in experienced centres, early surgical intervention is recommended in asymptomatic patients who exhibit one of these features (Class IIa, level of evidence B).

For patients who are unsuitable for surgery, M-TEER has emerged as a significant alternative treatment. Current recommendations support its use in symptomatic patients with severe PMR who are deemed at high or prohibitive surgical risk, provided the valve anatomy is suitable for transcatheter repair (class of recommendation IIb, level of evidence B) [14].

It’s crucial to note that delaying treatment of PMR can lead to left ventricular dilation, which creates a vicious cycle: the ventricle enlarges due to volume overload, which in turn worsens the MR by altering valve geometry. In other words, what begins as PMR can evolve into a mixed mechanism, where a functional mechanism is added due to changes in LV shape and function. This shift can reduce the chances of a durable repair and may limit the effectiveness of both surgery and M-TEER. Moreover, pharmacological therapy in severe PMR may provide transient symptomatic relief but can also obscure disease progression. Although agents such as diuretics, vasodilators, or rate-control drugs can alleviate HF symptoms, they do not halt the underlying structural deterioration of the MV or prevent adverse LV remodeling. Consequently, patients initially managed conservatively may miss the optimal window for surgical intervention. Nevertheless, postponing surgery until LV impairment is associated with increased risk of persistent postoperative LV dysfunction, heart failure, and reduced survival [17, 19, 20, 21].

**Key message**: Optimal timing and patient selection are critical in the management of PMR. While surgery remains the gold standard for symptomatic and select asymptomatic patients, M-TEER offers a valuable alternative for those at high surgical risk with favourable anatomy. Treatment delay, until undetected LV dysfunction occurs, can adversely affect both surgical and M-TEER outcomes.

### 3.3 WHICH DATA: Which Evidence Supports Our Choices?

Surgical repair remains the cornerstone of treatment for PMR, a position historically supported by the EVEREST II trial. However, these data must be interpreted considering advancements made since the study was conducted. The EVEREST II trial was conducted between 2005 and 2008, marking the early stages of M-TEER development. During this period, operator experience was limited, and only first-generation MitraClip systems were available.

Furthermore, the trial did not differentiate between subtypes of degenerative mitral disease—such as fibroelastic deficiency and BD—which are now known to influence procedural complexity, outcomes, and device suitability. Consequently, evidence from contemporary registries and newer device iterations is more representative of current clinical practice.

For instance, in the EXPAND (A Contemporary, Prospective Study Evaluating Real-world Experience of Performance and Safety for the Next Generation of MitraClip Devices), which employed third-generation MitraClip devices (NTR and XTR), patients with PMR, who represented approximately half of the study population, had a significantly lower all-cause mortality at one year (12.5%) compared to high-risk patients in the EVEREST II trial (23.8%) [22].

Moreover, in the EXPAND, 28% of patients had complex mitral valve anatomy, characterized by features such as a broad regurgitant jet, non-central jet location (outside the A2-P2 segment), multiple significant jets, small mitral valve area (<4.0 cm^2^), calcified landing zones, severely degenerative leaflets with large flail or prolapse, and limited leaflet tissue for device grasping. Despite these anatomical challenges, effective MR reduction was achieved in this subgroup, with 79.4% of patients reaching MR grade ≤1+ at 30-day follow-up [22].

This improvement was recently confirmed in the EXPAND G4 study, which evaluated the fourth-generation MitraClip XTW device. Among patients with PMR, 89% achieved MR grade ≤1+ at one year, compared to 31% in the EVEREST II trial and 85% with third-generation devices in the previous EXPAND [23].

Notably, the authors highlighted that these results were numerically comparable to those reported in two recent surgical mitral valve repair trials, in which 90% and 92% of surviving patients had MR ≤1+ at one year [24, 25].

Moreover, the all-cause mortality rate at one year was 8%, markedly lower than the 24% observed in the high-risk cohort of the EVEREST II trial [23].

The efficacy of M-TEER has also been confirmed with the PASCAL device. In the CLASP study, 2-year outcomes showed 94% survival and 97% freedom from heart failure hospitalization among PMR patients compared with 94% and 83% in the EVEREST, with 71% achieving MR ≤1+ and 100% achieving MR ≤2+, along with significant improvement in functional status [26].

Additionally, the CLASP IID randomized trial directly compared the PASCAL and MitraClip systems in prohibitive-surgical-risk patients. At one year, the PASCAL device demonstrated noninferiority to MitraClip in terms of safety and effectiveness, with 96% of patients achieving MR ≤2+ and over 77% achieving MR ≤1+, alongside sustained improvements in symptoms and quality of life [27].

However, specific aetiology appears to play a crucial role in procedural complexity and outcomes in PMR. As first demonstrated by Gavazzoni *et al*. [28], in a study specifically evaluating patients with BD undergoing MitraClip implantation, BD was associated with more complex anatomy, including bileaflet prolapse, multiscallop involvement, and fewer instances of chordal rupture compared to non-BD prolapse. While procedural success and safety were similar between BD and non-BD patients, the BD group required a greater number of clips and had longer procedure times. Additionally, optimal MR reduction (MR ≤1+) at discharge was less frequently achieved in BD patients (26% vs 42%), and durability at 3 years was lower, with only 62% maintaining MR ≤2+ compared to 80% in the non-BD group. Although overall mortality did not differ, there was a trend toward higher HF hospitalization in the BD cohort [28].

Furthermore, a flail mitral leaflet has been associated with favourable outcomes following MitraClip implantation. In the GIOTTO (Italian Society of Interventional Cardiology (GIse) registry Of Transcatheter treatment of mitral valve regurgitaTiOn) registry, a multicenter study involving 588 patients with significant PMR, patients with flail leaflets experienced a lower incidence of the composite endpoint—cardiac death and first rehospitalization for heart failure—at two years compared to those without flail leaflets (13% vs. 23%, *p* = 0.009). Moreover, multivariate analysis identified flail leaflet aetiology as an independent predictor of improved outcomes [29].

Interestingly, despite being younger, patients without flail leaflets showed higher mortality and rehospitalization rates during follow-up. This cohort presented with more advanced disease characteristics at baseline, including higher NT-proBNP levels, worse New York Heart Association (NYHA) functional class, and a higher prevalence of severe tricuspid regurgitation, all of which reflect more advanced MV pathology. These findings underscore again the importance of optimal timing for intervention. Patients with flail leaflets often present with localized severe disease, making them more suitable candidates for earlier intervention [29].

Collectively, these data emphasize the importance of tailoring transcatheter edge-to-edge repair strategies based on the underlying MV pathology. While BD presents procedural challenges and may lead to less durable outcomes, patients with flail leaflet pathology can achieve beneficial midterm results with M-TEER.

Currently ongoing randomized trials—including REPAIR MR (Percutaneous MitraClip Device or Surgical Mitral Valve Repair in Patients With Primary Mitral Regurgitation Who Are Candidates for Surgery; NCT04198870), PRIMARY (Percutaneous or Surgical Mitral Valve Repair; NCT05051033), and MITRA-HR (Multicentre Study of MitraClip in Patients With Severe Primary Mitral Regurgitation at High Surgical Risk; NCT03271762)—are designed to evaluate and compare the outcomes of M-TEER versus surgical mitral valve repair in both lower-risk and high-risk patient populations.

**Key message**: While surgical MV repair remains the gold standard for PMR, advances in M-TEER technology and operator experience have significantly improved outcomes, especially with newer-generation devices. Evidence shows that M-TEER is effective and safe in carefully selected patients, particularly those with flail leaflets, though more complex anatomies like BD present greater challenges and less durable results. Ongoing randomized trials will further clarify the optimal role of M-TEER versus surgery across risk profiles.

### 3.4 HOW: How do We Approach The Procedure?

**Fig. 3.**
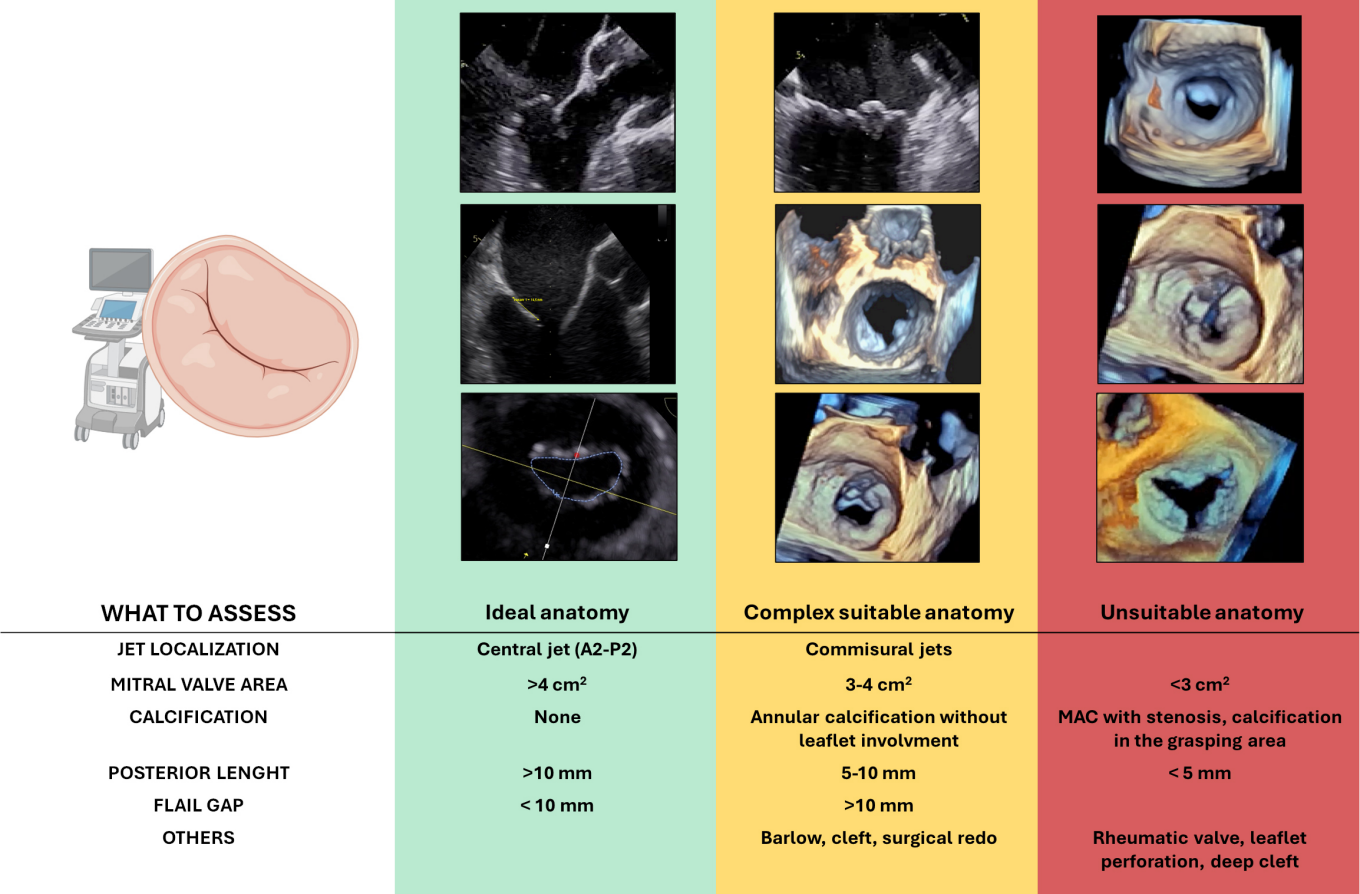
**Anatomical features relevant to patient selection for transcatheter edge-to-edge repair (M-TEER) in primary mitral regurgitation**. Based on echocardiographic assessment, anatomies are categorized as ideal (green), complex but suitable (yellow), or unsuitable (red) for TEER. Abbreviation: MAC, mitral annular calcification. Figure partially created with BioRender.

In the PMR approach with M-TEER, careful anatomical assessment is essential to determine procedural feasibility and predict procedural success (Fig. 3).

A thorough pre-procedural transesophageal echocardiographic (TOE) evaluation is critical. In order to evaluate candidates for M-TEER in PMR, the following anatomical features must be taken into consideration:

 - Central regurgitant jets (typically involving P2 or A2 segments) due to isolated leaflet prolapse represent the most favourable anatomy. In contrast, isolated commissural jets (A1/P1 or A3/P3) increase procedural difficulty, and multiple jets may complicate leaflet capture and result in suboptimal coaptation.

 - A posterior leaflet length >10 mm is ideal for effective grasping. A length of 7–10 mm is considered acceptable, though more challenging, while lengths between 5–7 mm pose significant technical difficulties. Leaflets <5 mm are generally considered unsuitable.

 - M-TEER is feasible when the flail gap is <10 mm and flail width is <15 mm. Larger flail segments increase procedural complexity and the risk of residual MR.

 - The absence of calcification, particularly in the grasping zone, is crucial for effective clip attachment and to ensure long-term durability. Annular calcification without leaflet involvement increases procedural complexity but is not a strict contraindication. However, calcification affecting the grasping zone of the leaflet precludes the technical feasibility of the procedure.

 - A baseline mitral valve area (MVA) >4.0 cm^2^ is ideal. An MVA between 3.5–4.0 cm^2^ may still be acceptable, but values <3.0 cm^2^ are generally unsuitable due to the increased risk of iatrogenic mitral stenosis.

 - BD, characterized by multi-scallop involvement, excessive leaflet tissue, and annular dilation, often makes it difficult to identify and treat a dominant regurgitant jet, resulting in technical challenges and uncertain durability.

 - Redo mitral valve surgery is not a contraindication, but altered leaflet morphology and the presence of prosthetic annuloplasty rings can impair clip manoeuvrability and leaflet grasping.

 - Clefts, especially deep ones and those involved in the MR mechanism, pose a considerable challenge. Despite the evolution of specific strategies for cleft repair, outcomes remain uncertain.

**Key message**: Successful M-TEER in PMR depends on careful anatomical evaluation; ideal candidates have central jets (typically P2/A2), adequate leaflet length (>10 mm), small flail gaps (<10 mm), absence of calcification in the grasping zone, and sufficient mitral valve area (>4.0 cm^2^). Complex anatomies—such as BD, multiple jets, commissural lesions, clefts, or prior surgery—pose greater procedural challenges and may reduce durability.

### 3.5 WHEN: What do We Know About Long-Term Outcomes?

Despite the increasing adoption of M-TEER for the treatment of PMR, robust long-term data remain scarce. Most of the available evidence derives from observational registries or RCTs with relatively short- to mid-term follow-up, often limited to procedural safety and 1-year clinical outcomes. This limitation leaves several important clinical questions unanswered, particularly regarding the long-term durability of MR reduction, the incidence of reintervention or recurrent MR, and how M-TEER compares to surgical repair in terms of survival and freedom from heart failure hospitalization.

Although EVEREST II included a 5-year follow-up, the study was conducted with early-generation devices and during a phase of limited operator experience. More recent prospective registries have provided additional insights, though most are limited by shorter follow-up durations and heterogeneous populations.

The GIOTTO registry includes a significant proportion of patients with PMR. At 2-year follow-up, in the entire population, all-cause mortality approached 35% and HF hospitalization rates were approximately 15%. Kaplan-Meier curves showed that patients with PMR, compared with SMR, presented a better outcome in terms of mortality and hospitalizations for HF [30].

Similarly, the MiTra-Ulm registry, which enrolled patients from a single high-volume center, reported a 3-year all-cause mortality rate of 36.6%, with no significant differences observed between aetiologies. Moreover, the authors highlighted that patients treated more recently had significantly lower mortality and major adverse cardiac and cerebrovascular event (MACCE) rates compared to those treated in the early phase of the program, underscoring the impact of technological advancements and increasing operator experience in improving outcomes [31].

Registries such as EXPAND and EXPAND G4 have included patients with both primary and secondary MR treated with newer-generation MitraClip devices. Although these studies have shown favourable safety profiles and outcomes, follow-up beyond 1–2 years remains limited.

The MitraSwiss registry, one of the largest prospective cohorts with stratified long-term data, reported a 5-year mortality of 45% in patients with PMR versus 54% in those with SMR. However, multivariable analysis showed that MR aetiology was not an independent predictor of death or major adverse cardiac event (MACE), which were instead driven by factors such as anaemia, renal dysfunction, and reduced LVEF [32]. Comparable results were observed in the TRAMI (Transcatheter Mitral Valve Interventions) registry, where the 4-year mortality reached 53%, supporting the concept that M-TEER, while effective, is often offered to high-risk patients with limited long-term survival due to advanced systemic disease [33].

However, stratified data by valve pathology (e.g., BD vs fibroelastic deficiency) are typically lacking.

In summary, while real-world registries have demonstrated the feasibility and safety of M-TEER in PMR with encouraging early outcomes, definitive long-term data, especially from RCTs, are still lacking.

**Key message**: Long-term data on M-TEER for PMR are limited, with most evidence from observational registries showing acceptable mid-term safety and outcomes but high mortality driven by patient comorbidities rather than valve pathology. While newer devices and growing experience improve results, definitive long-term durability and comparative effectiveness versus surgery remain uncertain, highlighting the need for further randomized trials with extended follow-up.

## 4. Secondary Mitral Regurgitation

### 4.1 WHAT: What Secondary MR are We Treating?

SMR (or functional mitral regurgitation) is not due to a primary structural abnormality of the MV apparatus, but rather to atrial or ventricular pathology, otherwise anatomically normal leaflets. It usually occurs because of adverse ventricular remodeling, most commonly in the setting of ischemic or non-ischemic dilated cardiomyopathy.

**Fig. 4.**
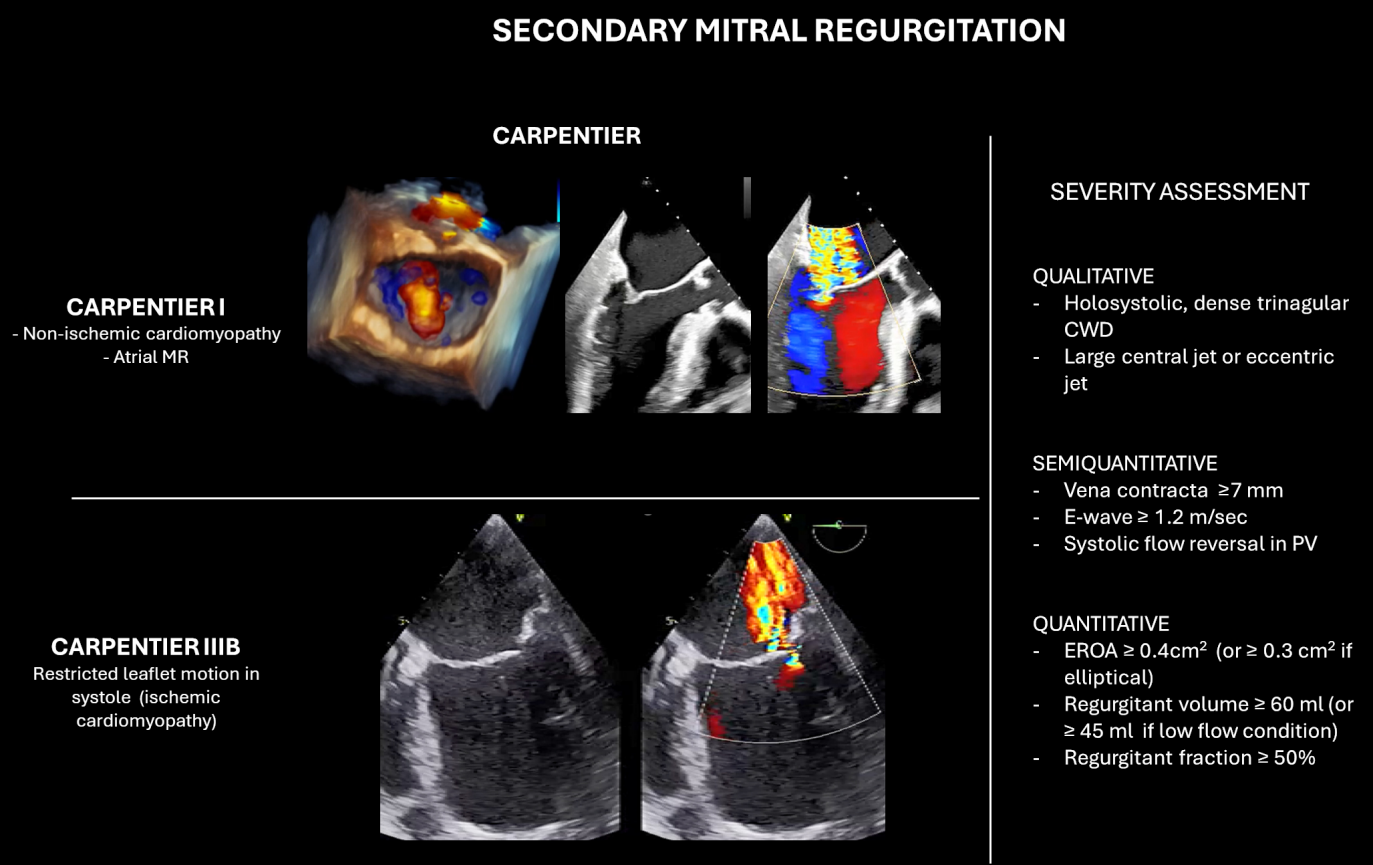
**Classification and echocardiographic severity assessment of secondary mitral regurgitation (SMR)**. The left panel illustrates Carpentier’s classification in SMR. The right panel summarizes qualitative, semi-quantitative, and quantitative parameters used for MR grading. *Abbreviations*: CWD, continuous wave Doppler; PV, pulmonary vein; EROA, effective regurgitant orifice area.

According to Carpentier’s classification (Fig. 4), Type I SMR is characterized by annular dilation. This form is frequently observed in patients with nonischaemic cardiomyopathy, where left ventricular remodeling and dilation lead to an enlarged mitral annulus and loss of annular contraction, ultimately preventing effective leaflet coaptation. Additionally, chronic atrial fibrillation (AF) with progressive left atrial enlargement can also cause annular dilation in the absence of left ventricular dysfunction. This entity is increasingly recognized as atrial SMR, a distinct subset of Type I SMR. Type IIIb SMR involves restricted systolic leaflet motion due to apical and posterior tethering and is typically seen in ischemic cardiomyopathy. Regional wall motion abnormalities, particularly in the inferior or posterior walls, lead to asymmetric displacement of the papillary muscles and tethering of the posterior leaflet, resulting in a posteriorly directed regurgitant jet. In patients with more diffuse myocardial dysfunction, such as multivessel coronary disease or advanced nonischaemic cardiomyopathy, a more symmetric tethering of both leaflets may occur, often producing a centrally directed MR jet.

Atrial SMR is increasingly recognized due to the higher prevalence of heart failure with preserved ejection fraction (HFpEF), where chronic atrial dilation (often due to longstanding atrial fibrillation) is the primary driver of MR. The typical anatomical changes include flattening of the mitral valve geometry and insufficient leaflet coaptation, often producing a central regurgitant jet. In approximately 20–30% of cases, posteriorly directed regurgitant jets are caused by atriogenic tethering. This occurs because, while the anterior mitral annulus is firmly anchored to the aortic root—a fixed and stable structure—the posterior annulus is attached to a more mobile and compliant region at the junction between the left atrium and the inlet of the left ventricle. As the left atrium and mitral annulus enlarge, the posterior annulus is displaced outward, over the crest of the LV inlet. This displacement causes tension and restricted motion of the posterior leaflet, resulting in functional tethering [34].

Ventricular SMR, on the other hand, is typically associated with heart failure with reduced ejection fraction (HFrEF) and is linked to both annular dilation and leaflet tethering due to adverse ventricular remodeling. This distinction is highlighted in the recently published European Guidelines on Valvular Heart Disease, which clearly differentiate between the two entities in terms of both diagnostic criteria and therapeutic implications [14]. In the setting of atrial SMR, management strategies primarily focus on rhythm and rate control, while the use of Sodium-Glucose Transport 2 (SGLT-2) inhibitors represents a key component of treatment in patients with HFpEF.

In ventricular SMR, by contrast, optimizing GDMT for HF is essential. Cardiac resynchronization therapy may also play a key role in improving MV mechanics in selected cases. However, while the benefit of M-TEER in patients with ventricular SMR is well-established based on several RCTs, the evidence supporting M-TEER in atrial SMR remains restricted to observational studies, which report high procedural success rates and a favourable safety profile [35, 36, 37].

In summary, understanding the diverse mechanisms underlying SMR is essential for tailoring effective and individualized treatment strategies in which mitral regurgitation is not merely a passive side-effect of disease severity, but a dynamic consequence and active contributor to the progression of primary cardiomyopathy.

**Key message**: SMR arises from atrial or ventricular pathologies rather than primary valve abnormalities. Atrial SMR, which is associated with left atrial dilation and frequently with HFpEF, involves annular dilation and atriogenic tethering. Conversely, ventricular SMR results from adverse left ventricular remodeling with leaflet tethering, which is commonly observed in HFrEF. The differentiation of these subtypes is of critical importance for the guidance of therapeutic approaches, as they differ in terms of their pathophysiology, prognosis, and therapeutic management.

### 4.2 WHO: Who Should We Treat?

In contrast to PMR, decision-making in SMR is more challenging, as MR frequently reflects progressive myocardial disease and it’s characterized by temporal variability in the severity of regurgitation, as it is strongly influenced by the patient’s hemodynamic condition. LV dimensions and function, symptom burden, and response to GDMT are crucial to determine candidacy for intervention.

According to the last European Guidelines on Valvular Heart Disease, M-TEER is recommended to reduce HF hospitalizations and improve quality of life in symptomatic patients with impaired LVEF (<50%) and persistent sever SMR without concomitant coronary artery disease, despite optimal GDMT and CRT if indicated (class of recommendation I, level of evidence A) [14]. Current guidelines also recommend considering M-TEER in patients who do not fulfil the specific clinical and echocardiographic criteria, for symptom improvement (class of recommendation IIb, level of evidence B) [14].

Methods for quantifying the severity of SMR are similar to those used for PMR, but with some important considerations unique to SMR. One major challenge arises from the impact of left LV dysfunction on the accuracy of color flow Doppler, which can lead to underestimation of MR severity. Moreover, the Proximal Isovelocity Surface Area (PISA) assumes an elliptical orifice that can underestimate the severity of MR. Therefore, lower thresholds of EROA ≥30 mm^2^ can be considered in SMR patients, as these values have been associated with improved prognosis following treatment [38]. Moreover, we can describe different temporal patterns according to MR etiology, resulting in corresponding changes in the PISA radius. Colour M-mode imaging and continuous wave Doppler profile are useful to detect these dynamic variations in MR flow during the entire cardiac cycle. In SMR, the EROA shows a phasic pattern, with early and late systolic peaks and a mid-systolic reduction, reflecting the rise in transmitral pressure that promotes more effective leaflet closure at mid-systole. Consequently, in SMR, EROA may be underestimated if the PISA radius is measured in mid-systole and overestimated if assessed in early or late systole [39].

In this context, the 3D Auto Color Flow Quantification (CFQ) application by Philips enhances the accuracy of RVol measurement by combining fluid dynamics with 3D colour flow imaging during TOE, enabling automated, quantitative MR assessment throughout systole. The validity of the software has been demonstrated in a series of studies, showing better agreement with cardiac magnetic resonance and lower variability compared to traditional 2D PISA methods, also highlighting the well-known differences in MR flow dynamics between degenerative and functional MR [40, 41].

### 4.3 WHICH DATA: Which Evidence Supports Our Choices?

The two pivotal trials, COAPT and MITRA-FR, delineate the longstanding debate between proportionate and disproportionate SMR because of completely divergent results.

We have already learned, first hypothesized by Grayburn *et al*. [42], the concept of proportionality in MR, whereby the severity of MR is assessed relative to the degree of LV dilation and dysfunction. In this context, the COAPT trial enrolled patients with severe MR but relatively less advanced LV dilation and dysfunction, a profile in which MR plays a disproportionately large role in haemodynamic profile and symptoms. In contrast, MITRA-FR included patients with more extensive LV remodeling, where MR was more likely a consequence rather than a primary contributor to heart failure. In such cases, the ventricle may be so severely damaged that correcting MR has minimal effect on the underlying disease trajectory.

This highlights a critical concept: not all secondary MR is equal. The success of M-TEER hinges upon identifying patients within the “therapeutic window”. These are patients with severe MR who still have left ventricular remodelling that is not too advanced to preclude the benefits of unloading.

The RESHAPE-HF2 trial introduces a distinct patient. While patients enrolled had similar age and comorbidities compared to COAPT and MITRA-FR, they were overall less ill, as reflected by lower natriuretic peptide levels and higher eGFR [11]. Moreover, this population exhibited a less severe MR, testified by a mean EROA of 0.23 cm^2^, against the 0.41 cm^2^ of COAPT and 0.31 cm^2^ of MITRA-FR [11]. However, the population is more comparable to the COAPT one, but with a smaller MR and similar left ventricular end-diastolic volume (LVEDV). The degree of MR could explain why no benefit was found in terms of mortality reduction, but only in HF hospitalization and symptoms.

In response to the findings, Gupta *et al*. [43] proposed a shift from the original proportionality model to a more pragmatic volume-based approach that aims to reconcile the divergent outcomes across the three major randomized trials. According to the authors, the lack of benefit in MITRA-FR may be attributed to extensive LV dilation (mean LVEDV = 252 mL), suggesting that MR was a consequence of advanced cardiomyopathy rather than a primary therapeutic target. In contrast, patients in COAPT had less LV dilation (mean LVEDV = 192 mL), which means less ventricular remodeling, but more severe MR, and accordingly experienced significant reductions in both HF hospitalizations and mortality.

RESHAPE-HF2 falls between these two extremes (mean LVEDV = 205 mL), and its outcomes reflect this intermediate profile: while M-TEER reduced HF hospitalizations, it did not impact mortality. The volume-based model preserves the importance of LV dimensions and MR severity, but it moves away from rigid proportionality calculations based on EROA-to-LVEDV ratios.

Moreover, consistent with these findings, the EXPANDed study demonstrated that patients with moderate SMR experienced similar clinical benefits to those with severe MR following MitraClip implantation [44]. 1-year outcomes were comparable across both groups in terms of MR reduction, LV reverse remodeling, improvement in functional status (NYHA class), quality of life (as measured by the Kansas City Cardiomyopathy Questionnaire), as well as all-cause mortality and heart failure hospitalizations [23].

This result emphasizes that the “one-size-fits-all” approach is no longer sufficient and reinforces the need for careful, phenotype-driven patient selection in M-TEER candidates.

Ultimately, this evolving understanding raises a key clinical question: is the current MR grading system adequate to identify SMR patients most likely to benefit from M-TEER, or should we adopt a more ventricular volume–based approach?

**Key message**: The divergent outcomes observed in the COAPT, MITRA-FR, and RESHAPE-HF2 studies underscore the heterogeneity of secondary MR, emphasising the necessity for a comprehensive, ventricular volume-based approach to identify patients who fall within the therapeutic window where M-TEER can provide the most significant benefits.

### 4.4 HOW: How do We Approach The Procedure?

In the case of M-TEER for SMR, evaluation should focus on a combination of:

- COAPT-derived clinical and echocardiographic selection criteria. To be considered for M-TEER, patients should fulfil the following:

- LVEF between 20% and 50%.

 - Left ventricular end-systolic diameter (LVESD) ≤70 mm.

 - Pulmonary artery systolic pressure (PASP) ≤70 mmHg.

 - Symptomatic heart failure (NYHA class II, III, or ambulatory IV) despite optimized GDMT.

 - No severe RV dysfunction.

 - At least one heart failure hospitalization within the previous year or increased natriuretic peptide levels.

- Anatomically suitable valve morphology for clip implantation. Key echocardiographic parameters for procedural feasibility specific to SMR include:

- A coaptation reserve, defined as the available leaflet tissue beyond the annular plane that can be approximated with the clip, greater than 3 mm, is generally associated with successful leaflet grasping and durable MR reduction. When the coaptation reserve is <3 mm, the risk of ineffective clip attachment and residual MR increases significantly, making the procedure technically more challenging and potentially less durable.

 - A tenting height of ≤11 mm suggests only moderate leaflet tethering and favourable geometry for clip approximation. When the coaptation depth exceeds this threshold, the increased tethering may impair adequate leaflet coaptation, reduce procedural success, and be associated with a higher likelihood of recurrent MR.

**Fig. 5.**
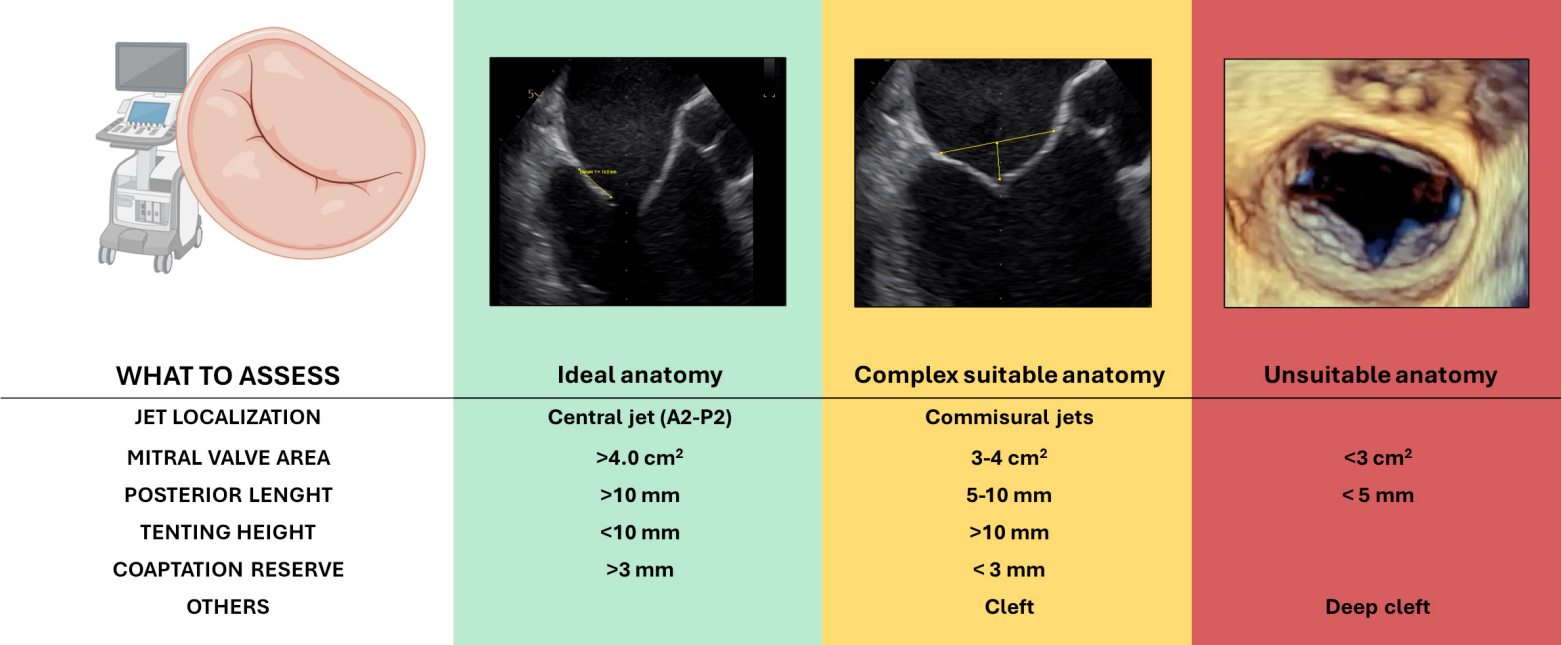
**Anatomical features relevant to patient selection for transcatheter edge-to-edge repair (M-TEER) in secondary mitral regurgitation**. Based on echocardiographic assessment, anatomies are categorized as ideal (green), complex but suitable (yellow), or unsuitable (red) for TEER. Figure partially created with BioRender.

However, many of the anatomical factors, including posterior leaflet length, MVA, and the presence of a cleft, which have been previously discussed in PMR, are also of significance in SMR (Fig. 5).

In particular, cleft-like indentation, especially between the P1–P2 and P2–P3 scallops, may be observed in patients with severe leaflet tethering or significant annular dilatation. These indentations can result in residual MR jets after M-TEER, particularly when the leaflet tissue is thinned in correspondence with the cleft. If the main regurgitant jet originates from such an indentation, a two-device strategy using the convergent clips technique can be attempted.

It is also important to consider that SMR is a highly preload-dependent valve disease. Anaesthesia-induced changes in loading conditions during the procedure may lead to a significant reduction in the severity of regurgitation, potentially affecting intraprocedural evaluation.

**Key message**: Successful M-TEER in SMR requires strict adherence to COAPT-like clinical criteria and detailed anatomical evaluation, with particular attention to leaflet tethering and coaptation reserve and tenting height.

### 4.5 WHEN: What do We Know about Long-Term Outcomes?

The COAPT trial remains the cornerstone study about long-term outcome with 5-year follow-up that confirmed the sustained benefit of MitraClip in patients with HF and severe SMR persisting despite GDMT. Compared with GDMT alone, M-TEER significantly reduced the rate of HF hospitalization (33.1% per year vs 57.2% per year; HR 0.53) and all-cause mortality (HR 0.72), with a low incidence of device-related complications (4 of 293 patients treated). Nonetheless, 5-year mortality remained high in both arms (57.3% with M-TEER vs 67.2% with GDMT), reflecting the progressive nature of HF in this population [38].

Importantly, 21% of control arm patients crossed over to receive MitraClip after two years, which may have attenuated between-group differences. Furthermore, the trial was conducted before the widespread use of Sodium–Glucose Cotransporter-2 (SGLT2) inhibitors. However, COAPT specifically enrolled patients with significant MR refractory to GDMT, and the persistent benefit observed suggests that M-TEER remains valuable even when contemporary pharmacologic therapy is optimized.

Several observational registries have also provided longer-term insights into real-world SMR populations. The MitraSwiss registry reported a 5-year mortality of 54% in SMR patients undergoing M-TEER, similar to those with PMR, with adverse outcomes driven by baseline comorbidities such as renal dysfunction and low LVEF rather than MR aetiology per se [32]. In the TRAMI registry, 4-year mortality similarly reached 54%, reinforcing the notion that long-term survival is often limited by underlying systemic disease rather than procedural efficacy alone [33].

Emerging data from next-generation device registries such as EXPAND G4, while promising in terms of procedural safety and early efficacy, still lack extended follow-up beyond 2 years, and heterogeneous inclusion criteria further complicate interpretation [23]. As in PMR, future studies are expected to clarify durability and survival benefit across different SMR phenotypes, particularly with the inclusion of patients treated under contemporary GDMT standards.

**Key message**: Long-term data, from the COAPT trial and observational studies, confirm the sustained benefit of M-TEER in SMR patients, with reduced HF hospitalizations and mortality up to 5 years.

## 5. Conclusion

In the M-TEER era, it is increasingly important not only to distinguish between primary and secondary MR but also to recognize and classify their respective subtypes, as these are associated with varying degrees of procedural complexity and clinical outcomes. Such distinctions should be incorporated into diagnostic evaluation, as well as in the design and interpretation of clinical trials. Furthermore, the optimal timing of intervention remains a matter of ongoing debate. In the specific context of SMR, particularly, delayed treatment is associated with a reduced clinical response, particularly in cases of advanced ventricular dilation. This emphasises the significance of timely intervention, ideally prior to the occurrence of substantial ventricular remodeling, which would otherwise result in the loss of effective unloading. In this context, the role of transcatheter mitral valve replacement will also need to be carefully integrated as a potential alternative in anatomically unsuitable cases for M-TEER.
